# *Cis* Interactions of Membrane Receptors and Ligands

**DOI:** 10.1146/annurev-cellbio-120420-103941

**Published:** 2023-06-20

**Authors:** Enfu Hui

**Affiliations:** Department of Cell and Developmental Biology, University of California, San Diego, La Jolla, California, USA

**Keywords:** *cis* interaction, *trans* interaction, receptor, ligand, signaling

## Abstract

Cell-cell communication is critical for the development and function of multicellular organisms. A crucial means by which cells communicate with one another is physical interactions between receptors on one cell and their ligands on a neighboring cell. *Trans* ligand:receptor interactions activate the receptor, ultimately leading to changes in the fate of the receptor-expressing cells. Such *trans* signaling is known to be critical for the functions of cells in the nervous and immune systems, among others. Historically, *trans* interactions are the primary conceptual framework for understanding cell-cell communication. However, cells often coexpress many receptors and ligands, and a subset of these has been reported to interact in *cis* and profoundly impact cell functions. *Cis* interactions likely constitute a fundamental, understudied regulatory mechanism in cell biology. Here, I discuss how *cis* interactions between membrane receptors and ligands regulate immune cell functions, and I also highlight outstanding questions in the field.

## 1. INTRODUCTION

### 1.1. What Are *Cis* Interactions and *Trans* Interactions?

*Cis* interactions in biology are not to be confused with the *cis* terminology in chemistry. In the context of cell biology, *cis* interactions and *trans* interactions are typically used to describe the orientation of an interaction between two membrane proteins with respect to the membranes they are anchored to. *Cis* interactions have the two interacting proteins anchored on the same membrane, and conversely, *trans* interactions have the two interacting proteins on two opposing cell membranes. Thus, a *cis* interaction often refers to cell-intrinsic interactions of two membrane proteins, while a *trans* interaction refers to binding between two proteins from different cells.

### 1.2. *Trans* Interactions in the Immune System

The immune system protects the host through both innate and adaptive mechanisms. Innate immunity includes natural killer (NK) cells, which destroy tumor or infected cells, and phagocytes [dendritic cells (DCs), macrophages, etc.], which engulf and digest foreign pathogens or dying cells. Some phagocytes can act as professional antigen presenting cells (APCs) through the surface display of peptide fragments derived from the engulfed substance via the major histocompatibility complex (MHC) (Neefjes et al. 2011). Adaptive immunity is mediated by B cells and T cells. B cells produce soluble antibodies to neutralize antigens in the body fluid. T cells have two general classes—CD8^+^ and CD4^+^. The former kill tumor or infected cells (target cells) upon physical contact, while the latter regulate other immune cells. Central to all immune cell function are molecular interactions between membrane receptors and ligands that often occur between two cells. Both B cell and T cell responses rely on antigen-specific receptors to recognize foreign or altered self molecules. T cell antigen receptor (TCR) interacts in *trans* with the MHC-bound peptide antigen, presented by a target cell or professional APC. The TCR signal is critically and reciprocally regulated by costimulatory and coinhibitory receptors, both of which are triggered by their ligands on the APCs (Chen & Flies 2013). These interactions collectively lead to the formation of the immunological synapse (IS) that determines the nature and quality of the immune response (Dustin 2014). Synaptic *trans* interactions are also central to the communication between an NK cell and target cell (Orange 2008), between a B cell and T cell (Mitchison 2004), etc. In the current paradigm, ligand:receptor *trans* interactions elicit signal transduction downstream of the receptor, thereby influencing the survival, metabolism, proliferation, differentiation, migration, and effector functions of immune cells. Some of these interactions have been successfully targeted for immunotherapies against a subset of human cancers (Baumeister et al. 2016, Waldman et al. 2020).

### 1.3. Why Should We Care About *Cis* Interactions?

While *trans* interactions are undeniably critical for cell-cell communication, cell-intrinsic *cis* interactions likely constitute a fundamental, overlooked regulatory mechanism in cell biology, for at least two reasons listed below.

#### 1.3.1. *Cis* interactions are likely quite common.

In a typical textbook image, a receptor on one cell interacts with a ligand on another cell in *trans*, while the possible *cis* interactions are peripheral to the scheme. However, immune cells coexpress many different receptors and ligands (Chen & Flies 2013), and a single cell can codisplay both receptors and their respective ligands (Keir et al. 2008). These membrane proteins can be intrinsically expressed or obtained from other cells. It is increasingly appreciated that lymphocytes acquire and redisplay APC-derived membrane ligands through trogocytosis (Joly & Hudrisier 2003), a contact-dependent cellular ingestion process conserved in mammals (Davis 2007, Nakada-Tsukui & Nozaki 2021). The fluidity of the cell membrane would allow for lateral diffusion of the receptor and ligand on the same membrane, and therefore, two proteins with intrinsic binding affinities could in principle interact in *cis*, unless they are spatially segregated or their interacting domains are constrained to a certain angle that prevents their binding. Therefore, it is reasonable to assume that *cis* interactions are quite common.

An important consideration for *cis* interactions is the binding geometry, as many ligand:receptor interactions occur in a head-to-head fashion, which appears to be optimized for *trans* interactions. Therefore, their *cis* interactions would require the deformation of the proteins and/or underlying membrane. In this sense, it is important to note that cell membranes are not flat but instead are covered by 3D membrane protrusions, including microvilli, lamellipodia, pseudopodia, and filopodia (Orbach & Su 2020). Membrane invaginations are also ubiquitous, as they are intrinsically associated with membrane trafficking, particularly endocytosis. Endocytosis-associated negative membrane curvature can promote *cis* interactions between CD28 and its B7 family ligands CD80 (B7-1) and CD86 (B7-2) (Zhao et al. 2023). We speculate that such negative membrane curvatures can provide a platform for *cis* interactions in general. Membrane proteins containing multiple ectodomains or a long stalk might be able to bend considerably to interact with their ligand or receptor in *cis* even on a flat membrane. For example, it was proposed that the inhibitory receptor Ly49A on NK cells bends considerably to interact with class I MHC (MHCI) in *cis* (Doucey et al. 2004). Moreover, the bent conformation of integrin Mac-1 interacts with sialylated FcγRIIA in *cis* (Saggu et al. 2018). Finally, there are proteins, such as PDL1 and CD80, that appear to be specialized for side-by-side parallel *cis* interactions, binding strongly in *cis* with few detectable *trans* interactions (Chaudhri et al. 2018, Zhao et al. 2019).

#### 1.3.2. *Cis* interactions can be physiologically significant.

*Cis* interactions can have important functional consequences. For example, *cis* interactions can interfere and compete with the canonical *trans* interactions to regulate cell-cell communication. This was illustrated in the case of *cis* HVEM:BTLA, *cis* PDL1:PD1, and *cis* PDL1:CD80 interactions, all of which inhibit one or more *trans* interactions (Chaudhri et al. 2018; Cheung et al. 2009; Sugiura et al. 2019; Zhao et al. 2018, 2019). Furthermore, mounting evidence suggests that at least some receptors can be activated by their ligands in *cis*, including Notch (Nandagopal et al. 2019), CD2 (Li et al. 2022, McArdel et al. 2016, Muhammad et al. 2009), and CD28 (Stephan et al. 2007, Zhao et al. 2023). *Cis*-interaction-mediated cell-autonomous signaling might be particularly important when the cells of interest are sparsely distributed and/or are surrounded by cells that lack the ligand, thereby limiting the chance of *trans* interactions.

### 1.4. Why Are *Cis* Interactions Understudied?

There are several factors that contribute to our limited knowledge of *cis* interactions. First, *trans*-interaction-based cell-cell communication is the impetus of the immune system. Many researchers might intentionally ignore *cis* interactions to simplify the conceptual framework because *cis* interactions would create disarray for the already complex immune signaling network. Second, for existing ligand:receptor pairs that are proven to interact in *trans*, their *cis* interactions might be deemed impossible as a result of overlooking potential cell membrane curvatures and protein bending. Plasma membranes are often considered flat despite the existence of curvatures in a real cell membrane—including protrusions (positive curvatures) and invaginations (negative curvatures). Third, assays to specifically measure *cis* but not *trans* interactions are not readily available to many laboratories. Finally, even though the existence of a *cis* interaction can be demonstrated, it can be challenging to study its functional consequence due to the difficulty of excluding the contribution from *trans* interactions.

In this review, I first discuss known *cis* interactions and their functional significance in the immune system. I then review methods for measuring *cis* interactions and finish by outlining future directions on this exciting topic.

## 2. *CIS* INTERACTIONS IN THE IMMUNE SYSTEM

**Figure 1:**
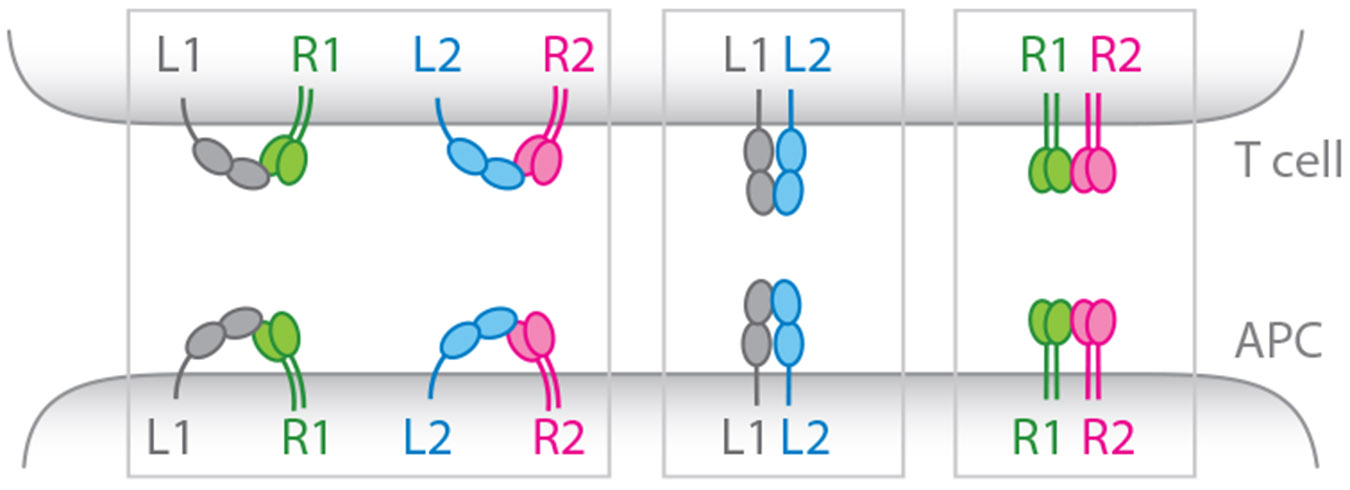
Three types of *cis* interactions. R1 and R2 denote two different receptors; L1 and L2 denote two different ligands. *Cis* interactions are illustrated in the context of T cells and APCs, but these interactions can occur in other types of immune cells and nonimmune cells. Moreover, while the *cis* interactions are depicted at the T cell:APC interface, they could in principle occur in nonsynaptic areas of the cell membranes. Abbreviation: APCs, antigen presenting cells.

Recent studies have independently demonstrated that some membrane receptors/ligands can interact both in *cis* and in *trans*, or only in *cis*. These *cis* interactions can occur in three ways: between a receptor and a ligand, between two ligands, and between two receptors (Figure 1). Below I describe the three types of *cis* interactions and their functional consequences. I do recognize that receptor and ligand terminology can be loosely defined and context dependent. In this review, I follow the commonly held view: A receptor refers to a membrane protein that is capable of mediating cell-intrinsic signaling through its intracellular domain; a membrane ligand refers to a membrane protein that binds and triggers the signaling of a membrane receptor. This article is not intended to review the total literature of *cis* interactions; rather I highlight studies that are most relevant to the topic.

### 2.1. Ligand:Receptor *Cis* Interactions

Although not universally appreciated, *cis* ligand:receptor interactions have been documented for several immunoreceptors. Earlier evidence of ligand:receptor *cis* interactions was somewhat indirect and was based on the finding that the binding of some immunoreceptors to their soluble or *trans* ligands is masked by coexpressed ligands. For example, CD22 (Siglec-2) expressed on resting B cells cannot capture α2-6 linked sialoside probes from solution unless the α2-6 sialic acids on B cells are destroyed (Collins et al. 2004, Razi & Varki 1998). Analogously, Doucey et al. (2004) reported *cis* interactions between MHCI and the inhibitory receptor Ly49A on NK cells based on the observation that coexpression of H-2Dd (MHCI) inhibited the ability of Ly49A transfectants to capture H2-Dd multimers. Although these competition experiments in a bulk cell population did not necessarily rule out the possibility that Ly49A on one cell engaged H2-Dd on another cell in *trans* to block the binding of H2-Dd multimers, Ly49A:MHCI *cis* interactions were later validated using more rigorous and sophisticated fluorescence correlation spectroscopy (Strömqvist et al. 2011). Moreover, several other *cis* ligand:receptor interactions have been demonstrated by Forster resonance energy transfer (FRET) (Cheung et al. 2009; Li et al. 2022; Masuda et al. 2007; Zhao et al. 2018, 2019, 2023).

**Figure 2:**
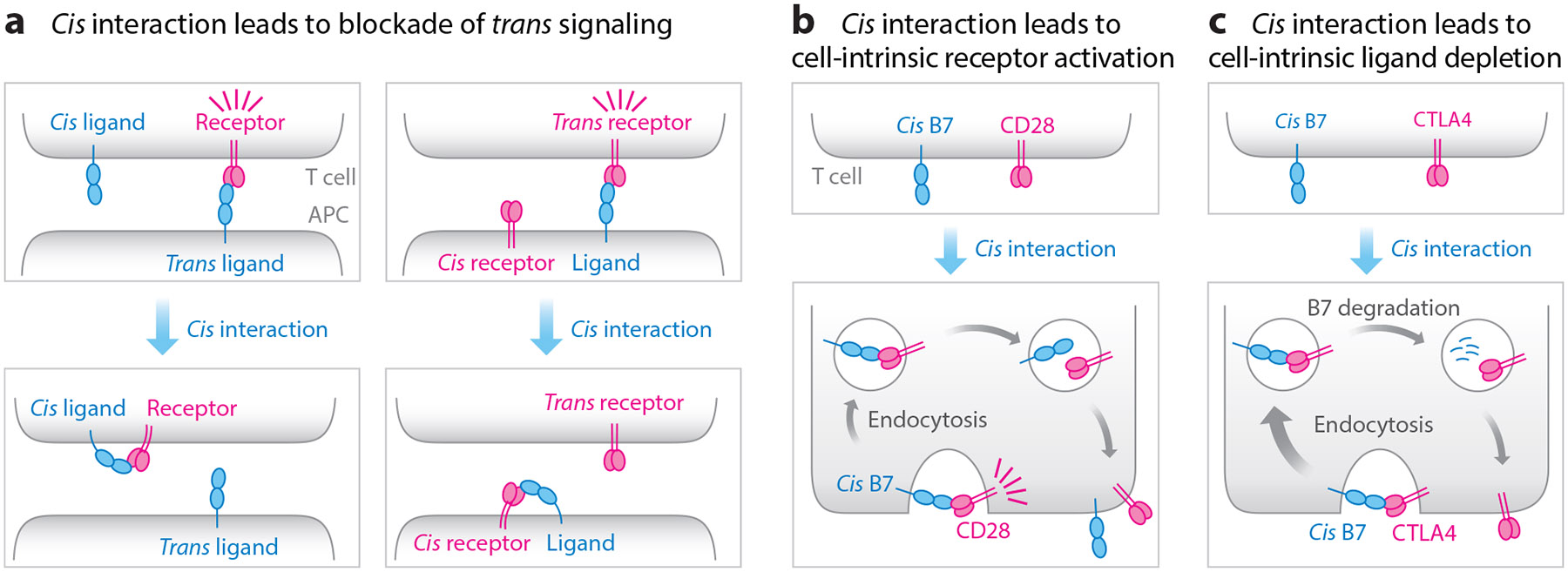
Functional consequences of ligand:receptor *cis* interactions. (*a*) When a cell coexpresses the receptor and its ligand, the *cis* ligand:receptor interaction can prevent the receptor from interacting with its *trans* ligand from a different cell. The *cis* BTLA:HVEM interaction falls into this category in which BTLA (ligand) blocks HVEM (receptor) in *cis* to prevent *trans* BTLA:HVEM signaling. This scenario may be the most relevant to circumstances in which the *cis* ligand is expressed at a higher level than the receptor. In a reciprocal scenario, a *cis* receptor serves to block its ligand, preventing its interaction with its *trans* receptor. This scenario may be the most relevant when the *cis* receptor is expressed at a higher level than the ligand. (*b*) Some *cis* ligand:receptor interactions can trigger cell-intrinsic signaling. For ligand:receptor pairs that interact in a head-to-head fashion, such as B7:CD28, their *cis* interactions can be promoted by negative membrane curvatures associated with endocytosis (Zhao et al. 2023). Following endocytosis, it is likely that CD28 and B7 dissociate due to the low pH in endosomes (Zenke et al. 2022), and are recycled back to the cell surface, poised for an additional round of *cis* signaling. (*c*) Some *cis* ligand:receptor interactions can lead to the internalization and degradation of the ligand. For example, the inhibitory immunoreceptor CTLA4 can act in *cis* to remove B7 (Xu et al. 2023) from the T cell surface and direct its lysosomal degradation of B7. This may allow CTLA4 to restrict T cell autostimulation through the B7:CD28 pathway. Abbreviation: APC, antigen presenting cell.

At a functional level, *cis* ligand:receptor interactions can have at least three consequences. First, *cis* interactions can block the more established *trans* ligand:receptor interactions, thereby attenuating the *trans* signaling (Figure 2a). Specifically, the *cis* H-2Dd:Ly49A interaction blocks the *trans* H-2Dd:Ly49A interaction to restrict inhibitory signaling through Ly49A (Doucey et al. 2004). Likewise, the *cis* PDL1:PD1 interaction on EL4 lymphoma cells prevents PDL1 from engaging PD1 on T cells. This, in turn, renders T cell–mediated in vitro killing of EL4 cells insensitive to PD1 blockade because the *trans* PDL1:PD1 signaling is pre-blocked by the *cis* interaction (Zhao et al. 2018). The net functional effect of *cis* BTLA:HVEM interactions is more complex because *trans* BTLA:HVEM interactions trigger bidirectional signaling, stimulatory through HVEM and inhibitory through BTLA. Cheung et al. (2009) showed that the *cis* BTLA:HVEM interaction represses HVEM-dependent NF-κB activation by blocking the *trans* BTLA:HVEM interaction, but the extent to which this *cis* interaction interferes with BTLA inhibitory signaling is unclear and likely depends on the relative amounts of BTLA and HVEM.

Second, some *cis* ligand:receptor interactions can induce cell-intrinsic signaling (Figure 2b). This appears to be the case for CD2 and its ligands, CD48 in mice and CD58 in humans, which are coexpressed by both T cells and APCs. The well-established *trans* CD2:CD48/CD58 interactions promote T cell activity through orchestrating multiple signaling axes (Demetriou et al. 2020). *Cis* CD2:CD48/CD58 interactions reportedly promote T cell activation by facilitating the recruitment of TCR signaling components (Li et al. 2022, McArdel et al. 2016, Muhammad et al. 2009), though the effects observed in these studies could in principle be mediated by *trans* CD2:CD48/CD58 interactions between T cells. Stephan et al. (2007) suggested that CD80 (B7-1) and 4-1BBL transduced to T cells can activate their receptors CD28 and 4-1BB in *cis*. Along this line, we recently showed that T cell B7 ligands (CD80 and CD86) can activate CD28 in *cis* to promote the survival, migration, cytokine production, and antitumor activity of CD8^+^ T cells (Zhao et al. 2023). The ability of *cis* B7:CD28 interactions to stimulate CD28 in a T cell–intrinsic fashion could provide onboard costimulation for T cells and bypass the requirement of B7-expressing professional APCs, which are relatively sparse in peripheral tissues.

How do *cis* interactions activate the receptor? This is still incompletely understood. However, the mechanism by which *trans* interactions activate the receptor is much better studied. *Trans* interactions would drive the ligand-receptor complexes to the cell-cell interface, where other ligated receptors are present. According to the kinetic segregation model (Davis & van der Merwe 2006), phosphorylation-dependent immunoreceptor signaling is restricted by the abundant and bulky transmembrane phosphatases CD45 and CD148. *Trans* ligand:receptor interactions from an opposing cell often create a tight membrane junction that excludes the bulky phosphatases to promote receptor phosphorylation by Src family kinases. Such a mechanism cannot be easily envisioned for *cis* signaling, as *cis* interactions could in principle occur anywhere on the plasma membrane as long as the conformation of the *cis* complex matches the membrane geometry. However, we recently found that *cis* B7:CD28 signaling occurs primarily at the IS, known to be a focal point of endocytosis (Griffiths et al. 2010), and appears to localize at the base of narrow membrane tubules driven by phosphatidylinositol-3-kinase (PI3K) and the membrane remodeling protein sorting of nexin 9 (SNX9) (Zhao et al. 2023). Indeed, an endocytosis-deficient CD28 mutant, which cannot interact with PI3K or SNX9, can be activated only by *trans* B7 but not by *cis* B7. These data suggest that endocytosis-associated negative curvatures might serve to enrich *cis* B7:CD28 complexes to the IS for productive signaling. Moreover, as an alternative but nonexclusive mechanism, *cis* B7:CD28 binding can promote the detachment of the CD28 intracellular domain (ICD) from the plasma membrane, presumably rendering it more accessible for kinases and effectors, as previously shown in the *trans*-signaling format (Dobbins et al. 2016).

Third, some *cis* ligand:receptor interactions can result in the internalization and degradation of the ligand (Figure 2c). This was recently shown for T cell inhibitory immunoreceptor CTLA4, which can act in *cis* to deplete B7 ligands from the T cell surface, thereby restricting B7:CD28 stimulatory signaling (Xu et al. 2023). This cell-intrinsic action of CTLA4 is consistent with its predominantly intracellular localization. Moreover, in the context of regulatory T cells, CTLA4-mediated *cis* endocytosis of B7 can act downstream of CD28-mediated B7 trogocytosis to deplete B7 from APCs (Xu et al. 2023). This mechanism might allow CTLA4 to exert cell-extrinsic effects suggested by in vivo experiments (Bachmann et al. 1999).

In summary, *cis* ligand:receptor interactions can have at least three biological consequences: competing with *trans* ligand:receptor interactions to attenuate *trans* signaling, triggering cell-intrinsic signaling through the receptor, and inducing ligand degradation (Figure 2).

### 2.2. Ligand:Ligand *Cis* Interactions

*Cis* interactions are not restricted to ligand:receptor pairs—they can also occur between membrane ligands. The homodimerization of membrane ligands, demonstrated in many cases, can be broadly considered *cis* interactions that increase the avidity of receptor binding. Here, I focus on *cis* interactions between two different ligands, as exemplified by the recently demonstrated PDL1:CD80 *cis* interaction (Chaudhri et al. 2018, Zhao et al. 2019). Because PDL1 is a PD1 ligand and because CD80 is a shared ligand for costimulatory immunoreceptor CD28 and coinhibitory immunoreceptor CTLA4, the *cis* PDL1:CD80 interaction has the potential of interfering with three signaling axes.

The PDL1:CD80 interaction was initially discovered by Butte et al. (2007), based on the micromolar affinity between their extracellular immunoglobulin variable (IgV)-like domains. Since PDL1 and CD80 are expressed by both APCs and antigen-experienced T cells, the PDL1:CD80 interaction was initially presumed to occur in *trans* to trigger bidirectional signaling through the ICDs of PDL1 and CD80. One of the first clues of the existence of the *cis* PDL1:CD80 interaction came from the finding that CD80 coexpression renders PDL1-positive tumor cells more susceptible to T cell–mediated killing (Haile et al. 2011). However, it was not until recently that the *cis* interaction was independently demonstrated by a split luciferase assay and FRET assays both in cells and in membrane reconstitution systems (Chaudhri et al. 2018, Zhao et al. 2019). These studies also provided evidence for the lack of *trans* PDL1:CD80 interactions. Thus, the PDL1:CD80 pair represents an interesting example of two proteins in the Ig-like superfamily interacting only in *cis* but not in *trans*. This strict *cis*-interacting modality is perhaps due to the structural rigidity of these two proteins. Functionally, the disruption of *cis* PDL1:CD80 interactions on DCs through point mutations or anti-PDL1 antibodies promotes tumor growth and suppresses autoimmunity in mouse models (Oh et al. 2020, Sugiura et al. 2019), demonstrating the physiological significance of this *cis* interaction.

**Figure 3:**
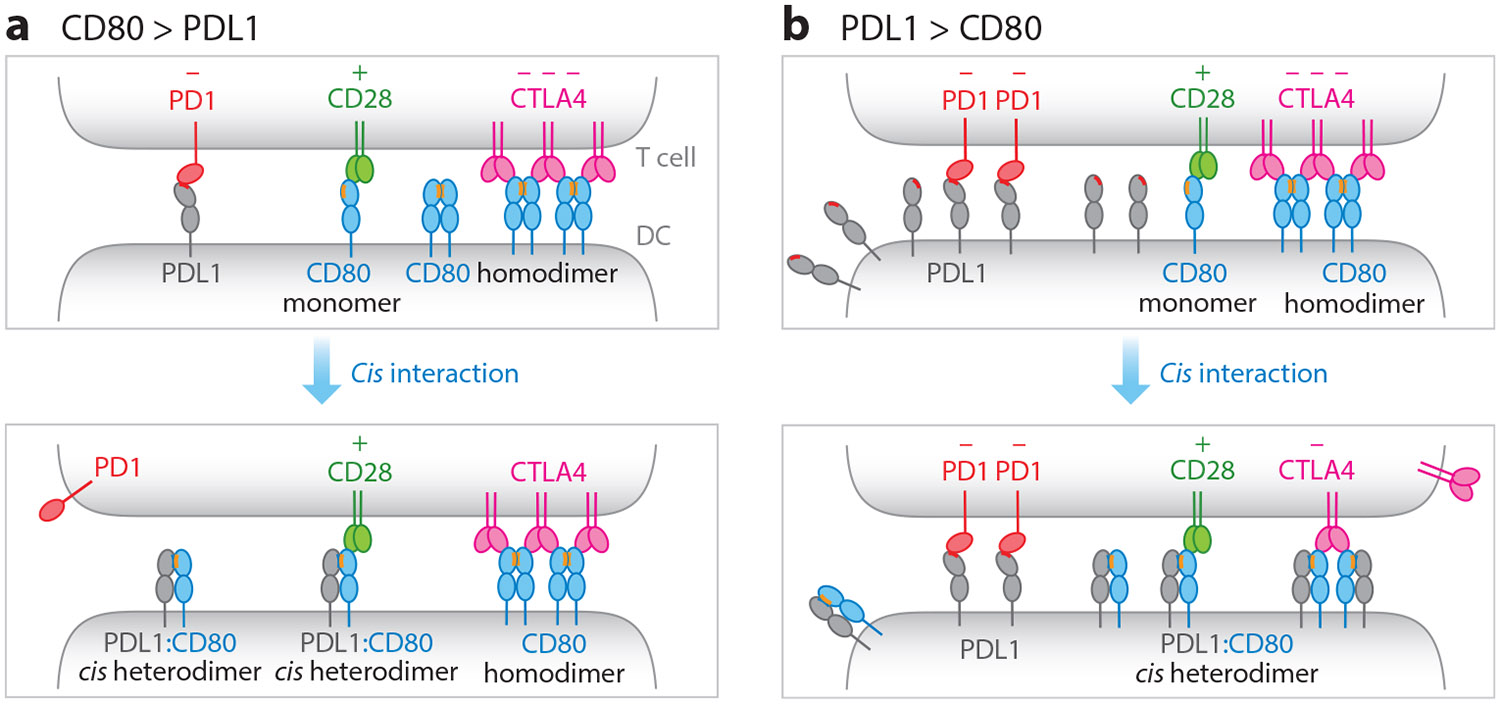
Regulation of PD1, CD28, and CTLA4 axes through the *cis* PDL1:CD80 interaction. Prior to the discovery of the *cis* PDL1:CD80 interaction, DCs were thought to display PDL1, the CD80 monomer, and CD80:CD80 homodimers, which *trans* interact with PD1, CD28, and CTLA4, respectively. The discovery of the *cis* PDL1:CD80 interaction suggests the existence of PDL1:CD80 heterodimers on DCs, and depending on the relative expression levels of PDL1 and CD80, the *cis* interaction could have a differential effect on PD1 and CTLA4 signaling. (*a*) When CD80 is in large excess of PDL1, PDL1 exists largely as the PDL1:CD80 heterodimer and is unable to bind PD1. However, the PDL1:CD80 heterodimer can bind and activate CD28. The excess CD80 molecules can exist as homodimers and interact with CTLA4 with a high avidity. (*b*) When PDL1 is in large excess of CD80, CD80 exists largely as PDL1:CD80 heterodimers at the expense of CD80:CD80 homodimers. PDL1:CD80 heterodimers similarly act as CD28 ligands but not PD1 ligands. Even though the PDL1:CD80 heterodimer can bind to CTLA4, it does so through a lower avidity than CD80:CD80 homodimers. Thus, CTLA4 activity is mitigated. Even though the PDL1:CD80 heterodimer cannot trigger PD1 signaling, the excess PDL1 can do so. A plus sign denotes stimulatory signaling and a minus sign denotes inhibitory signaling. Abbreviation: DCs, dendritic cells.

Mechanistically, *cis* PDL1:CD80 interactions on APCs regulate the relative strength of three signaling pathways in T cells: the costimulatory CD28 axis, the inhibitory CTLA4 axis, and the inhibitory PD1 axis. *Cis* PDL1:CD80 interaction inhibits both PDL1:PD1 and CD80:CTLA4 interactions but preserves CD80:CD28 interactions. *Cis* PDL1:CD80 interactions prevent PDL1 from binding and triggering PD1 signaling (Sugiura et al. 2019, Zhao et al. 2019) (Figure 3a). This is because the CD80 binding site and PD1 binding site on PDL1 partially overlap, allowing CD80 to physically block the PDL1:PD1 interaction (Chaudhri et al. 2018, Sugiura et al. 2019). Chaudhri et al. (2018) identified several point mutations in the PDL1 IgV domain that reduce both PD1 and CD80 binding. Sugiura et al. (2019) showed that CD80 binding requires the C strand of PDL1 IgV, which is also part of the PD1-binding face on PDL1 according to the PDL1:PD1 cocrystal structure (Lin et al. 2008).

The monovalent CD28:CD80 interaction proceeds through the opposite face as that of the PDL1:CD80 interaction (Evans et al. 2005, Ikemizu et al. 2000, Maurer et al. 2022, Sugiura et al. 2019); thus the *cis* PDL1:CD80 interaction does not affect the CD80:CD28 interaction (Figure 3a). Indeed, when we conjugated a CD28-positive, PD1-negative T cell with a PDL1/CD80 double-positive APC, all three proteins (CD28, CD80, and PDL1) accumulated to the T:APC interface leading to CD28 signaling (Zhao et al. 2019). This result suggests that CD28, CD80, and PDL1 form a tripartite complex to activate CD28. While PDL1 does not affect the CD80:CD28 interaction, it does inhibit the CD80:CTLA4 interaction through an avidity effect rather than a blocking effect (Zhao et al. 2019). This is because a covalent dimeric CTLA4 binds to noncovalent CD80 homodimers to form a multivalent array, based on their cocrystal structure (Stamper et al. 2001), but *cis* PDL1 disrupts CD80 homodimerization to decrease the avidity of the CD80:CTLA4 interaction (Zhao et al. 2019) (Figure 3b).

**Figure 4:**
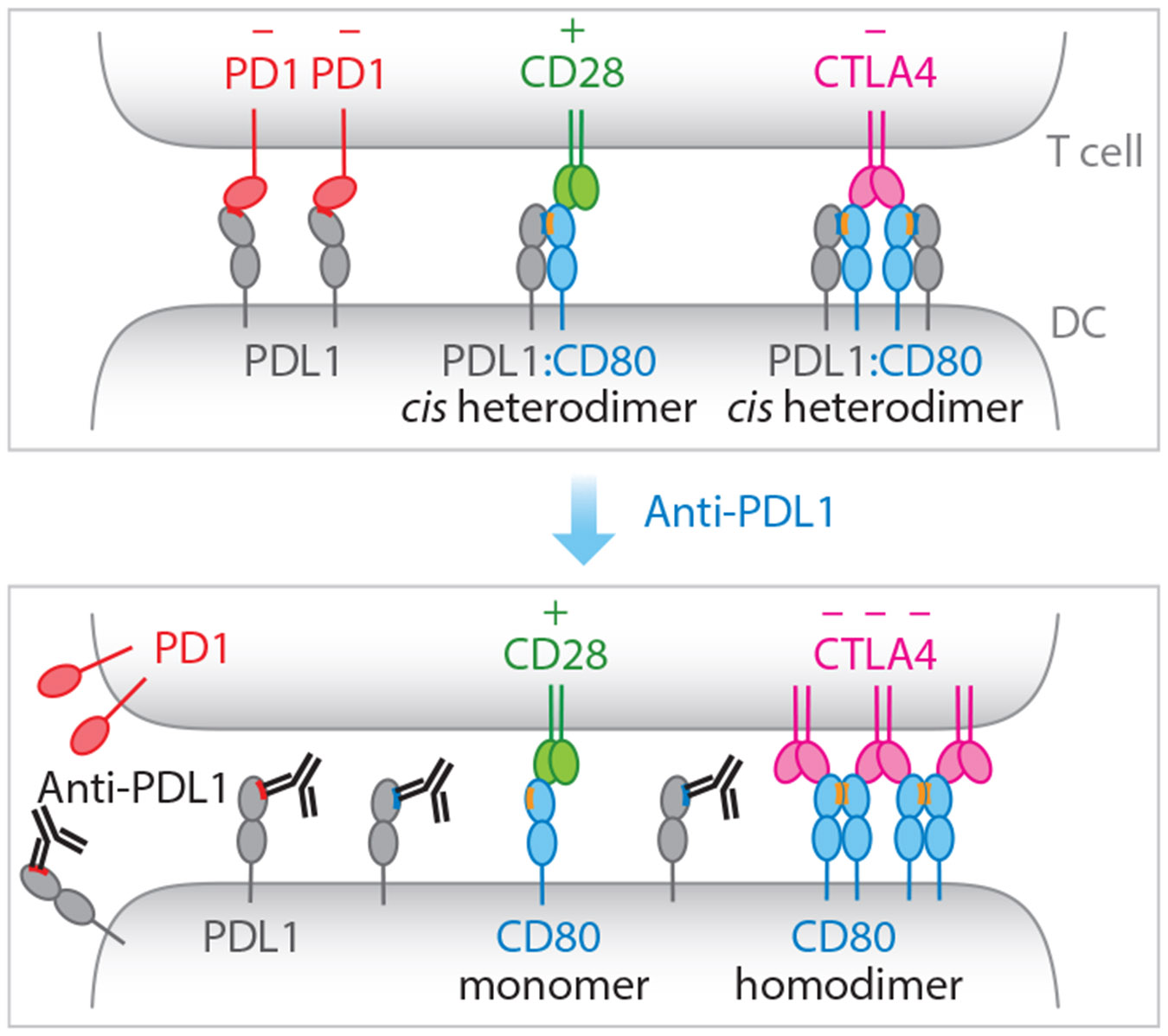
Dual blocking effects of therapeutic anti-PDL1. In the absence of anti-PDL1, isolated PDL1 triggers PD1, and *cis* PDL1:CD80 heterodimers bind CD28 and CTLA4. Administration of anti-PDL1 disrupts both *trans* PDL1:PD1 interaction and *cis* PDL1:CD80 interactions, creating isolated CD80 monomers and homodimers, the latter of which interact more strongly with CTLA4 due to an increase in avidity. Abbreviation: DC, dendritic cell.

Collectively, *cis* PDL1:CD80 interactions promote T cell immunity by repressing two inhibitory pathways (PD1 and CTLA4) while preserving the stimulatory signaling through CD28. The relative abundance of PDL1 and CD80 likely dictates whether *cis* PDL1:CD80 interactions primarily regulate PD1 or CTLA4. When CD80 is in excess of PDL1, *cis* PDL1:CD80 interactions serve to block PDL1:PD1 interactions (Figure 3a); conversely, when PDL1 is in excess of CD80, *cis* PDL1:CD80 interactions disrupt CD80 dimerization to mitigate CD80:CTLA4 interactions (Figure 3b). Notably, the US Food and Drug Administration–approved anti-PDL1 blockade antibodies atezolizumab, durvalumab, and avelumab block both PDL1:PD1 and *cis* PDL1:CD80 interactions. Blockade of the cis interaction promotes CD80 homodimerization and CTLA4 activity (Figure 4), leading to an immunosuppressive side effect for these antibodies (Zhao et al. 2019).

These studies revealed that both CD80 and PDL1 are bifunctional ligands. CD80 not only acts as a ligand for CD28 but also blocks PDL1:PD1 signaling. PDL1 not only functions as a ligand for PD1 but also restricts CTLA4 activity by disrupting CD80 homodimers. The *cis* PDL1:CD80 heterodimer represents a novel form of the CD28 ligand, especially on professional APCs that express more PDL1 than CD80 such that monomeric and homodimeric CD80 is titrated out by *cis* PDL1. The various forms of CD80 and their receptor binding activities are summarized in Table 1. Finally, while PDL1 and CD80 represent the best characterized *cis*-interacting ligand pair, other ligand:ligand *cis* interactions likely exist and similarly mediate molecular cross talk between multiple signaling pathways.

### 2.3. Receptor:Receptor *Cis* Interactions

Many immunoreceptors exist as homodimers, either covalently (constitutively) such as CD28 and CTLA4 or noncovalently (transiently), which presumably increase the avidity of their interaction with the ligand and/or downstream effectors. In other cases, different immunoreceptors can heterodimerize to form a functional complex. These can be conceptually considered as *cis* interactions. One of the best studied examples involves the family members commonly found on innate immune cells, where they recognize microbe-associated molecular patterns (MAMPs) (Medzhitov 2001). The ectodomains of TLR exhibit a characteristic horseshoe fold comprising multiple leucine-rich repeats (Bell et al. 2003). Dimerization creates a binding pocket for MAMPs. While many TLRs function as homodimers, several work as heterodimers, which expand the ligand spectrum of TLRs (Farhat et al. 2008). For example, TLR2 heterodimerizes with TLR1 or TLR6, and its *cis*-interacting partner dictates its MAMP specificity (Ozinsky et al. 2000). The TLR2:TLR1 *cis* heterodimer recognizes triacyl lipopeptides derived from mycoplasma or gram-negative bacteria. In contrast, the TLR2:TLR6 *cis* heterodimer recognizes diacyl lipopeptides derived from mycoplasma and gram-positive bacteria (Akira et al. 2001).

*Cis* heterodimerization has also been reported for Ig superfamily immunoreceptors. Johnston et al. (2014) reported that the stimulatory immunoreceptor CD226 associates with the inhibitory immunoreceptor TIGIT in *cis*, based on a time-resolved FRET assay. They also provided evidence that *cis* TIGIT:CD226 interactions disrupt CD226 homodimerization. However, whether TIGIT and CD226 bind directly or indirectly has not been formally proven. Future studies are needed to elucidate the structural mechanism, membrane geometry requirement, and functional consequence of *cis* TIGIT:CD226 interactions. It is possible that *cis* TIGIT:CD226 interactions decrease the avidity of CD226 signaling. Alternatively, but nonexclusively, the *cis* interaction might serve to bring the TIGIT and CD226 signalosomes into close proximity and promote their biochemical cross talk. If one considers both BTLA and HVEM as receptors, the reported *cis* HVEM:BTLA interaction (Doucey et al. 2004) is another example of receptor *cis* heterodimerization. Finally, several SLAM family receptors undergo either homophilic or heterophilic interactions (Cao et al. 2006, Engel et al. 2003). Although these receptors have been largely studied in the context of *trans* interactions, their *cis* interactions are likely owing to their extensive coexpression on hematopoietic cells.

## 3. APPROACHES TO MEASURING *CIS* INTERACTIONS

It has been challenging to measure pure *cis* interactions due to the difficulty in distinguishing *cis* versus *trans* interactions in cell cultures. Here I summarize several established approaches for detecting and characterizing *cis* interactions on the same membrane.

### 3.1. Cell-Based FRET Assays

FRET, which entails energy transfer between two spectrally overlapping fluorophores that are within a distance of 5–10 nm, has been successfully used to detect *cis* interactions. In the absence of FRET, excitation of the donor (high-energy) fluorophore would lead to emission of only the donor fluorescence but not the acceptor (low-energy) fluorophore. In the presence of FRET, the excited donor fluorophore transfers part of its energy to the acceptor, leading to acceptor emission and a concomitant decrease of the donor fluorescence. Accordingly, there are many strategies to measure FRET but three of them have been particularly useful for cell biologists: sensitized emission, acceptor photobleaching, and fluorescence lifetime imaging microscopy. All three FRET modalities have been applied for measuring *cis* interactions.

#### 3.1.1. Sensitized emission.

Sensitized emission, also referred to as two-color ratiometric imaging, is a process in which the donor fluorophore is excited by a specific wavelength and the emission of the donor and acceptor is collected via filters. This method may be the most straightforward based on the FRET concept, but it can be limited by the cross talk between fluorophores and the requirement of extensive controls. Both microscopy and flow cytometry–based sensitized emission have been used to detect *cis* interactions (Cheung et al. 2009, Masuda et al. 2007).

#### 3.1.2. Acceptor photobleaching.

Acceptor photobleaching, also known as donor dequenching, measures the increase (dequenching) in donor fluorescence upon acceptor photobleaching. This method is straightforward, quantitative, and performed using only a single sample. Using this method, we have provided evidence of *cis* interactions between PDL1 and PD1 (Zhao et al. 2018), between PDL1 and CD80 (Zhao et al. 2019), and between CD28 and its B7 ligands (CD80 and CD86) (Zhao et al. 2023). However, this method also has limitations, since photobleaching is irreversible and can only be done once per cell.

#### 3.1.3. Fluorescence lifetime imaging microscopy.

Fluorescence lifetime is the duration a fluorophore spends in the excited state before emitting a photon and returning to its ground state (Berezin & Achilefu 2010). The lifetime is an intrinsic property of a fluorophore independent of its concentration, laser intensity, scattering, etc., but it depends on environmental factors such as the presence of a quencher (Lakowicz 1999). Since the acceptor of a FRET interaction essentially acts as a quencher of the donor, the fluorescence lifetime of the donor fluorophore can be used to measure FRET efficiencies. Fluorescence lifetime imaging microscopy (FLIM) is more reliable than ratiometric measurements because it is less prone to acceptor interference, but its application on *cis* interactions has been rare, perhaps due to its more specialized, sophisticated, and expensive instrument. Notably, however, Saggu et al. (2018) used FLIM to examine *cis* interactions between sialylated FcγRIIA and the integrin Mac-1.

#### 3.1.4. Measurement modes of cell-based FRET assays.

In FRET applications the fluorescence can be measured using either flow cytometry or microscopy. Flow cytometry–based FRET has been applied to demonstrate the existence of HVEM-BTLA *cis* interactions (Cheung et al. 2009). This approach allows for an unbiased examination of a cell population due to its high-throughput nature, but it requires a careful design of controls to correct for fluorescence cross talk in the case of sensitized emission. Moreover, if the fluorophores are conjugated to the ectodomains of the proteins of interest, additional controls are required to rule out contributions from *trans* interactions. Thus, it seems ideal to have the fluorophores conjugated to the ICDs. However, ICDs are often far away from the *cis* interaction sites located in the ectodomains. Therefore, the lack of ICD FRET cannot be used to rule out a *cis* interaction.

Microscopy-based FRET has also demonstrated its utility in detecting *cis* interactions (Li et al. 2022; Masuda et al. 2007; Saggu et al. 2018; Zhao et al. 2018, 2019, 2023). The advantage of this approach is that it allows researchers to measure FRET for individual, isolated cells, thereby ruling out contributions from *trans* interactions. Notably, these recent studies all measured FRET based on acceptor photobleaching. In principle, the sensitized emission could also be viable but would need more controls. The main disadvantage of microscopy-based FRET is its low-throughput nature. Therefore, it is important to unbiasedly and carefully analyze many individual cells to reach a conclusion.

### 3.2. Split Luciferase Complementation Assay

Split luciferase assays rely on the ability of two inactive halves of a luciferase to reconstitute to an active enzyme upon their physical proximity. This system is composed of a large fragment and a small complementary fragment that interact with a very low affinity, and hence their association only occurs in close proximity. To detect interactions between two proteins of interest (A and B), the large and small fragments of the luciferase are fused to A and B, respectively, and cotransfected to cells. When A and B associate, the two fragments complement to form an active luciferase, which produces luminescence in the presence of a substrate. This method was used to demonstrate *cis* interactions between PDL1 and CD80 (B7-1) (Chaudhri et al. 2018).

A split luciferase assay is sensitive and straightforward, as the signal is detected by a luminescence microplate reader. It also allows for measurement of protein-protein interactions in live cells. However, it suffers similar limitations as the flow cytometry–based FRET assay when detecting *cis* interactions due to its bulk format. It is often desirable to fuse the luciferase fragments to the ICDs of the proteins of interest because, in this configuration, only *cis* but not *trans* interactions can lead to reconstitution of the enzyme. However, this strategy might generate false negatives because, as mentioned above, some head-to-head *cis* interactions have their ICDs pointing in the opposite directions. Such *cis* interactions can be detected by fusing the split luciferases to the extracellular domains (ECDs) of proteins of interest, but to avoid false positive results, careful control conditions must be run to rule out contributions from *trans* interactions. Moreover, some proteins cannot tolerate the large luciferase fragment, trapping the fusion protein in the endo-plasmic reticulum (Zhao et al. 2023). Therefore, caution must be taken to check if the luciferase fusion disrupts the target protein localization in cells.

### 3.3. Fluorescence Cross-Correlation Spectroscopy

Fluorescence correlation spectroscopy can determine both the concentration and diffusion coefficient of a fluorescently labeled biomolecule, through measuring the emission intensity fluctuation as the molecules diffuse through a focused light. On this basis, dual-color fluorescence cross-correlation spectroscopy (FCCS) was developed to detect biomolecular interactions by simultaneously measuring the two molecular species (A and B) that are labeled with spectrally separated fluorophores. The diffusion of a dual-color A:B complex produces synchronized (correlated) signal fluctuations, whereas the single-color unbound species (A or B) creates independent fluctuations. Thus, dual-color FCCS can determine the concentrations and diffusion coefficients of all three species: A, B, and the A:B complex. It was initially developed to detect and characterize molecular interactions in solution (Schwille et al. 1997). Strömqvist et al. (2011) further used it to examine the *cis* interaction between Ly49A and MHCI. The limitations of FCCS include its requirement of considerable mobilities of the molecules of interest and its inability to detect low-affinity interactions (Bacia & Schwille 2007). As an increasing number of facilities have now implemented FCCS, it should prove its value in future studies on *cis* interactions.

### 3.4. Membrane Reconstitution Assays

Cell-based measurements provide evidence for protein-protein proximity or codiffusion. However, they might not be sufficient to prove the existence of direct interactions since the two proteins of interest might be cross-bridged by a third molecule. Thus, membrane reconstitution of purified proteins is a complementary method to prove the existence of direct *cis* interactions between two proteins. Moreover, because the concentrations of the proteins can be precisely controlled, membrane reconstitution can also yield quantitative insights into *cis* interactions, e.g., 2D affinities. We recently combined membrane reconstitution, FRET, and microscopy to examine the quantitative nature of *cis* PDL1:PD1 interactions (Zhao et al. 2018) and *cis* PDL1:CD80 interactions (Zhao et al. 2019). However, there are several limitations with this method. First, the lipid composition of the reconstituted membranes often differs considerably from that of the biological membranes. This might in turn affect the behaviors of the proteins. Second, due to the intrinsic difficulty in the reconstitution of membrane proteins with controlled stoichiometry and orientation, our current membrane reconstitution method attaches the polyhistidine ECD or ICD of proteins to membranes through chelating lipids. These truncated proteins coupled to nickel lipids might not precisely reflect the native orientation of the transmembrane protein. However, we have found that liposomes attached with PDL1 can be captured by a supported lipid bilayer (SLB) attached with PD1 but not by an SLB attached with CD80 (Zhao et al. 2019). This finding is consistent with the notion that PDL1 and CD80 interact only in *cis* but not *trans*, demonstrating the utility of membrane reconstitution in examining geometry-sensitive interactions. Therefore, while cell-free reconstitution cannot match many features in cells, it provides the 2D membrane geometry and biophysical precision for identifying *cis* interactions. Nevertheless, it is important to cross-check findings from the membrane reconstitution system in cells. It is also important to note that planar lipid bilayers formed on a coverslip lack the curvatures found in a real cell membrane, and they therefore might not be able to reveal *cis* interactions that require membrane curvatures. In this sense, giant unilamellar vesicles lacking a solid support might be a better platform for the reconstitution of *cis* interactions.

Finally, all the abovementioned fluorescence-based measurements of *cis* interactions require the fusion of the protein of interest with a fluorescent protein, which might interfere with the *cis* interactions or even the folding/localization of the protein. One potential alternative approach is to use immunostaining to label the proteins of interest, but antibody binding can often interfere with *cis* interactions, by either blocking the binding interface or crosslinking the protein to increase the avidity of the interaction. To avoid these caveats, nonblocking monovalent fragment antigen-binding, single-chain variant fragments, or camelid-derived nanobodies can be used. Alternatively, nonblocking antibodies can be used to label the proteins of interest after cell fixation, but this prevents measurement on live cells. Therefore, caution must be taken in designing and interpreting data of *cis* interactions. It is often desirable to prove a *cis* interaction through multiple independent approaches.

## 4. APPROACHES TO MEASURING THE FUNCTIONAL CONSEQUENCE OF *CIS* INTERACTIONS

Several independent recent studies on *cis* PDL1:CD80 interactions have established a paradigm for dissecting the functional consequence and mechanistic basis of ligand:ligand *cis* interactions. The finding that PDL1 and CD80 interact only in *cis* but not in *trans* (Chaudhri et al. 2018, Zhao et al. 2019) has simplified the complexity of the signaling network. However, it is still quite challenging to study because *cis* heterodimerization can in principle affect at least three receptors: the PDL1-binding receptor PD1 and the CD80-binding receptors CD28 and CTLA4. Focusing on the effect on the PD1 axis, Sugiura et al. (2019) identified point mutations that selectively disrupt *cis* PDL1:CD80 interactions without affecting PDL1:PD1 interactions. This has allowed them to generate knock-in mice that lack *cis* PDL1:CD80 interactions specifically on DCs. They showed that disruption of this *cis* interaction on DCs promotes tumor growth and suppresses autoimmunity, establishing its physiological significance. This was corroborated by Oh et al. (2020), who reported enhanced tumor growth in mice upon treatment of an anti-PDL1 antibody that specifically disrupts PDL1:CD80 interactions without affecting PDL1:PD1 interactions. Focusing on the mechanistic basis, we combined quantitative biochemistry and cell lines with varying ratios of PDL1 and CD80 to show that *cis* PDL1:CD80 interactions inhibit both PDL1:PD1 and CD80:CTLA4 interactions but preserve CD80:CD28 interactions (Zhao et al. 2019). Thus, *cis*-interaction-disrupting drugs, mutations, and quantitative biology have proven their value in determining the functional consequence of ligand:ligand *cis* interactions.

To study the functional consequence of *cis* ligand:receptor interactions, there is an added challenge in excluding the contribution from *trans* interactions. For example, when determining the functional impact of T cell–intrinsic *cis* interactions of two proteins of interest, even if one uses APCs that lack both proteins, these two proteins could interact in *trans* between T cells. Therefore, any functional outcomes observed upon disrupting an interaction could be due to either a *cis* or a *trans* effect. It is therefore most desirable to identify point mutations or drugs that selectively disrupt *cis* but not *trans* interactions, or vice versa. However, such mutations or drugs are often challenging to identify. PDL1:PD1 *cis* interactions appear to occur through the same face as their *trans* interaction because (*a*) the crystal structure of a PDL1:PD1 IgV complex revealed only one binding interface, (*b*) *cis* PD1 and *trans* PD1 bind competitively to PDL1, and (*c*) mutations that disrupt the *trans* interaction also inhibit the *cis* interaction. To prove the functional significance of *cis* B7:CD28 interactions, we utilized a PI3K-binding-deficient mutant of CD28, which is defective in *cis* signaling but intact in *trans* signaling (Zhao et al. 2023). In the in vitro setting, *cis* signaling could be studied at the single-cell resolution within microchambers (Kobayashi et al. 2022) or in the presence of excess filler cells that lack both the ligand and receptor to prevent *trans* interactions (Xu et al. 2023, Zhao et al. 2023).

## 5. OUTSTANDING QUESTIONS

### 5.1. Are There Membrane Proteins That Interact Only in *Trans* but Not in *Cis?*

Among the many pairs of immunoreceptors and ligands that interact in *trans*, only a small subset was shown to also interact in *cis*, including BTLA:HVEM, PD1:PDL1, CD2:CD48/CD58, and CD28:CD80/CD86. However, the very short list of *cis* interacting pairs is likely due to the lack of robust approaches for detecting *cis* interactions or the reluctance to study *cis* interactions. Based on our data that B7:CD28 *cis* interactions occur in a head-to-head fashion at negatively curved membranes (Zhao et al. 2023) and given that cell membranes contain many local curvatures, we speculate that most, if not all, ligand:receptor pairs that are known to interact in *trans* can also interact in *cis* upon their coexpression. In this sense, some of the *cis* interactions can be considered local *trans* interactions promoted by negative membrane curvatures. Therefore, it might be interesting to ask the question of whether there are membrane protein pairs that interact only in *trans*, but not in *cis*. Identification of such obligatorily *trans*-interacting pairs might form the basis for engineering novel receptors that signal only in *cis* or only in *trans* to precisely modulate our immune system.

### 5.2. When and Where Do *Cis* Interactions Take Place?

At present, *cis* interactions are assumed to occur constitutively at the plasma membrane. However, given our recent finding that *cis* B7:CD28 signaling occurs at invaginated membranes of the IS (Zhao et al. 2023), it will be intriguing to determine the spatiotemporal dynamics of *cis* interactions. Superresolution microscopy should prove powerful in this effort. It is likely that proteins with structural flexibility can engage *cis* interactions at relatively flat membranes, but more rigid proteins might only *cis* interact at invaginated membranes. The subcellular location and timing of *cis* interactions might correlate with their functional outcomes.

### 5.3. Are There Long-Range *Cis* Interactions Between Membrane Protrusions?

Immune cells are covered by dynamic, 3D membrane projections such as microvilli, which are known to enrich certain receptors and signaling proteins (Cai et al. 2017, Ghosh et al. 2020, Orbach & Su 2020, Razvag et al. 2018). If these membrane projections are flexible enough to approach one another or bend back to the cell body, it may allow for *cis* interactions. Future studies are needed to determine the existence and functional consequence of such long-range *cis* interactions.

### 5.4. *Cis* Signaling Versus Endocytosis

While receptor endocytosis is known as a mechanism to downregulate *trans* ligand:receptor signaling (Sorkin & von Zastrow 2009), we recently showed that CD28 endocytosis promotes *cis* B7:CD28 signaling, and vice versa, to form a positive loop (Zhao et al. 2023). This raises the question of the generality of the endocytosis–*cis* signaling coupling: Is all *cis* signaling promoted by endocytosis? If so, does it share the same set of molecular machinery? Do the ligand and receptor remain bound during endocytosis? At what point do they dissociate? Do they signal from the endomembrane compartments?

### 5.5. What Is the Fate of *Cis* Complexes?

The fate of the *cis* signaling complex appears to be receptor dependent. In the case of *cis* B7:CD28 signaling, it is likely that both B7 and CD28 are endocytosed and then recycled back to the cell surface. In contrast, *cis* B7:CTLA4 interactions cause internalization and CTLA4-directed lysosomal degradation of B7 (Xu et al. 2023). This ligand-depleting action of CTLA4 is consistent with its extraordinarily endocytic property and allows it to restrict the display of costimulatory information on the immune cell surface. Little is known about the fate other *cis* signaling complexes, such as CD2:CD48/CD58 and 4-1BB:4-1BBL. The mechanism controlling recycling versus degradation during *cis* signaling is unknown but could be informed by such mechanisms in *trans* signaling.

### 5.6. Do All *Cis* Interactions Trigger Cell-Autonomous Signaling?

*Cis* interactions between a ligand and receptor should compete with their *trans* interactions to inhibit *trans* signaling unless *cis* and *trans* binding occurs through a nonoverlapping face. On the other hand, there are also reports that some *cis* interactions are sufficient to trigger cell-intrinsic signaling, including the CD2:CD48/CD58 pair (Li et al. 2022, McArdel et al. 2016, Muhammad et al. 2009) and B7:CD28 pair (Zhao et al. 2023). Do all *cis* interactions trigger signaling? This does not seem to be the case since no inhibitory signaling has been detected for MHCI:Ly49A *cis* interactions (Doucey et al. 2004). What are the molecular features for a signaling-capable *cis* interaction? Future studies are needed to define the principle for predicting the functional consequence of a *cis* interaction.

### 5.7. What Are the Similarities and Differences Between *Cis* and *Trans* Signaling?

It is now known that at least some *cis* interactions are sufficient to trigger cell-autonomous signaling. However, a fundamental unresolved question is whether and how *cis*-interaction-mediated signaling differs from *trans*-interaction-mediated signaling. Does *cis* signaling induce unique functional outcomes? At the cellular level, *cis* and *trans* signaling might differ in their downstream signaling pathways and subcellular localizations. At the tissue level, *cis* and *trans* signaling might operate at different anatomical sites and under different physiological settings. This can depend on where the ligand is expressed and on the cell densities. It is likely that *trans* signaling predominates in tissues where cell:cell contact is frequent. In contrast, *cis* signaling might play important roles in tissues lacking *trans* ligands or where the cell of interest is sparsely distributed.

## 6. CONCLUDING REMARKS

In summary, *cis* interactions are an important yet largely overlooked dimension in cell signaling. A deeper understanding of the role of *cis* interactions among the ligands and receptors on immune cells will likely inform potential side effects of the current therapeutic antibodies, as well as identify biomarkers on the heterogeneity of patients’ immune response. While this review focuses on *cis* interactions on immune cells, they are likely ubiquitous and critically regulate the fate and functions of cells. Recent studies have established several robust methods for detecting, characterizing, and perturbing *cis* interactions both in vitro and in vivo. These studies have laid the groundwork for many exciting discoveries on *cis* interactions down the road.

## Figures and Tables

**Table 1:** Receptor binding activities of PDL1 and CD80 and their dimeric forms

Form of ligands	PD1 binding	CD28 binding	CTLA4 binding
PDL1 monomer	+	−	−
CD80 monomer	−	+	+
CD80:CD80 homodimer	−	+	+++
PDL1:CD80 heterodimer	−	+	+
